# What You See Is What You Get? Exclusion Performances in Ravens and Keas

**DOI:** 10.1371/journal.pone.0006368

**Published:** 2009-08-05

**Authors:** Christian Schloegl, Anneke Dierks, Gyula K. Gajdon, Ludwig Huber, Kurt Kotrschal, Thomas Bugnyar

**Affiliations:** 1 Konrad Lorenz Forschungsstelle für Ethologie, Grünau im Almtal, and Department of Behavioural Biology, University of Vienna, Vienna, Austria; 2 Zoologisches Institut und Museum, Ernst-Moritz-Arndt Universität Greifswald, Greifswald, Germany; 3 Department of Neurobiology and Cognition Research, University of Vienna, Vienna, Austria; 4 Konrad Lorenz Institute for Ethology, Austrian Academy of Science, Vienna, Austria; University of Lethbridge, Canada

## Abstract

**Background:**

Among birds, corvids and parrots are prime candidates for advanced cognitive abilities. Still, hardly anything is known about cognitive similarities and dissimilarities between them. Recently, exclusion has gained increasing interest in comparative cognition. To select the correct option in an exclusion task, one option has to be rejected (or excluded) and the correct option may be inferred, which raises the possibility that causal understanding is involved. However, little is yet known about its evolutionary history, as only few species, and mainly mammals, have been studied.

**Methodology/Principal Findings:**

We tested ravens and keas in a choice task requiring the search for food in two differently shaped tubes. We provided the birds with partial information about the content of one of the two tubes and asked whether they could use this information to infer the location of the hidden food and adjust their searching behaviour accordingly. Additionally, this setup allowed us to investigate whether the birds would appreciate the impact of the shape of the tubes on the visibility of food. The keas chose the baited tube more often than the ravens. However, the ravens applied the more efficient strategy, choosing by exclusion more frequently than the keas. An additional experiment confirmed this, indicating that ravens and keas either differ in their cognitive skills or that they apply them differently.

**Conclusion:**

To our knowledge, this is the first study to demonstrate that corvids and parrots may perform differently in cognitive tasks, highlighting the potential impact of different selection pressures on the cognitive evolution of these large-brained birds.

## Introduction

Despite a recent increase of interest in exclusion performance (EP), relatively little is known about its prevalence in non-human animals (see [1] for a review). In a choice task, EP is given if one alternative is selected by excluding the alternative option [2]. Typically, an animal is confronted with a choice between two options A and B. Prior to choosing, it is informed that one option, say B, is the incorrect choice, and the choice would be exclusion-driven if the animal selects A over B. However, the mechanism on which this is based is unclear and may also differ between experiments [2], [3] and species [1], [4]. If spontaneously shown (i.e., without any evidence for learning), EP may be explained most easily (and cognitively least demanding) by avoidance of the wrong option [4]. In this case, nothing needs to be known about A, as the choice is solely based on knowledge about B. Alternatively A is inferred to be the correct solution because B is not. Here, both options are evaluated comparatively and the role of A is inferred. This mechanism has been labelled “inference by exclusion” [2], [3] or “reasoning by exclusion” [5].

Originally, EP was studied either with matching-to-sample and comparable procedures [4], [6]–[8] or with experiments designed to test language-trained animals: in the latter case, researchers were interested in whether the animals would be able to identify and learn the meaning of new words or signs via exclusion [9], [10]. In general, such tasks have been criticised for their artificiality [11] and more natural test setups have been advocated instead [12], [13]. Call and co-workers introduced two food-finding experiments [3], [14] which may fulfil that criterion [15]. In one of these tasks the animals are confronted with two bowls and food is hidden under one of them [3], [5]. In the crucial condition, the subjects are informed about the content of the empty bowl before they are allowed to choose; hence, by exclusion, they should avoid this bowl and choose the other bowl instead. In a second line of experiments, food is hidden in transparent or opaque tubes and the subjects are allowed to look into the tubes before they make their choice [14], [16], [17]. To exhibit EP, they have to choose the second tube without looking into it, after having first looked into the empty tube. This slightly more complicated setup allowed researchers to investigate further aspects: in some trials, the animals were aware of the position of the food, whereas in others they were not. It had been suggested that an adjustment of searching effort according to what is known may indicate that the animals are aware of their own state of knowledge ([14], [16], [17]; but see [15], [18] for a sceptical evaluation of the data). Finally, straight and bent tubes have been used to evaluate whether the subjects would appreciate the visual access given by differently shaped tubes; i.e. by looking into a straight tube from one side, the entire content of the tube is visually accessible, whereas the same is not true for a bent tube, as some content maybe hidden behind the bend. This task has been found to be surprisingly difficult for three- and five-year old children [19], and capuchin monkeys failed entirely [17].

While the mechanisms of EP are currently under debate, little is known about the prevalence and evolutionary history of EP in non-human animals; that is, which species are capable of EP and why [1]? Beside the great apes and chimpanzees *Pan troglodytes* in particular, which have been tested in a number of experiments [2], [3], [6], [8], [14], [20], [21], only two monkey species (Capuchin monkeys *Cebus apella* [17], [22], [23] and Tonkean macaques *Macaca tonkeana* [24]), dogs *Canis familiaris* [4], [5], [9], [25], sea lions *Zalophus californianus* [10], [26] and Bottlenose dolphins *Tursiops truncatus* [27] have been studied systematically. In birds however, only ambiguous evidence exists for pigeons *Columba livia* [4], [7] and anecdotal evidence for grey parrots *Psittacus erithacus* [28].

As the avian counterparts of the large-brained mammals, corvids and parrots exhibit similar cognitive abilities [29]–[31]. If the evolution of the cognitive abilities of mammals and birds led to similar cognitive skills, corvids and parrots may be seen as prime candidates for EP in birds. In consequence, we tested keas *Nestor notabilis*, and Common ravens *Corvus corax* in two exclusion tasks.

A direct comparison between these species may be a first step towards elucidating the trajectories of the evolution of avian cognition. Two opposing theories exist: the “adaptive specialisation hypothesis” argues that each species may possess very specific cognitive abilities in adaptation to its socio-ecology [12], [32]. Alternatively, a “general process view” proposes a broader set of cognitive abilities as a consequence of the evolution of large brains [33]. At the moment, arguments can be put forward for either process: on the one hand, corvids and parrots apparently do not differ in their abilities to solve means ends-tasks [34]–[37] and, within the corvids, tool-using New Caledonian crows *Corvus moneduloides* and non tool-using rooks *Corvus frugilegus* both solve a tool-related trap-tube task in a comparable way [38]–[40]; on the other hand, food-caching ravens and non-caching jackdaws *Corvus monedula* differ in observational spatial memory capacities [41] and highly social pinyon jays *Gymnorhinus cyanocephalus* differ from less social scrub jays *Aphelocoma californica* in the performance in transitive inference tasks [42].

To keep the results of the two species comparable to earlier studies conducted with primates [3], [17], [23] and dogs [5], we chose test paradigms similar to the ones introduced by Call and co-workers [3], [14], because they are simple and – as food finding-tasks – less artificial than matching-to-sample procedures [12]. In a similar approach capuchin monkeys demonstrated EP only in a task in which food was hidden under bowls, but not if food was hidden in tubes [17], [22], [23]. This contrasts not only previous findings in chimpanzees [3], [14] but further indicates that these two tasks may not be fully equivalent. Therefore, we used versions of both tasks in this study.

In our first experiment, birds had to search for food in two opaque tubes; using a bent and a straight tube we manipulated the visibility of food inside the tubes and studied the search patterns applied. This allowed us to investigate not only EP, but also whether the birds would appreciate the visual access given by differently shaped tubes. In a second experiment, the birds had to choose between one of two bowls [2], [3] having been given prior information about the content of one of the bowls.

## Materials and Methods

### Experiment 1: Food Hidden in tubes

#### Subjects

We employed eight two-year old, hand-raised ravens (four males, four females), which were kept in a 230 m^2^ - outdoor aviary in the Cumberland game park, Grünau im Almtal, Austria. The aviary was composed of three outdoor sections and five small testing compartments that were visually isolated from the other parts of the aviary. The outdoor parts of the aviary contained natural vegetation, bushes, conifer trees providing shade as well as rocks and logs. Indoor compartments had a floor of fine-grained sand and a few perches. If not experimentally tested, birds were allowed to range freely in the entire complex. Prior to this study, the birds had participated in a number of experiments, e.g. on gaze following [43], the use of gaze cues in object-choice tasks [44], [45] and visual perspective taking (Bugnyar, subm.; in this task, the ravens had to judge whether another raven could see a human caching food).

The ten keas were kept in a 150 m^2^ - outdoor aviary at the Konrad Lorenz Institute for Ethology in Vienna, Austria. The group consisted of four hand-raised, three-year old males and six parent-reared males (4–8 years old), which all hatched in captivity. Two additional birds (one male and one female) were present throughout testing but did not participate in the experiments. The aviary was composed of three compartments (each 10 m×5 m and 4 m high), and was equipped with logs, granite blocks, perches, ponds and wooden shelters. Various toys as part of other investigations were regularly exchanged. The floor consisted of fine-grained sand. One compartment of the aviary could be visually isolated for experimental testing. When not being tested, birds were allowed to move freely in the entire aviary. Prior to this study, these birds had been used in a variety of tests, among them tests on string-pulling [37], cooperation and social learning [46], [47].

#### Testing procedure

The ravens were tested in late summer/autumn 2006 and the keas were tested in spring 2007. All birds were tested by CS. Birds were not food-deprived, but were not tested subsequent to feedings. Food from prior feedings was potentially available in food caches (ravens) or freely distributed throughout the aviary (keas). Water was available ad libitum. To keep birds motivated, we used highly favoured food rewards which were not available outside the experimental context: for ravens, we used pieces of cheese, commercial dog food or cereals (depending on individual preferences), for keas we used half peanuts. The size of the food rewards was comparable for all birds and they were clearly motivated to obtain the rewards.

All birds were tested individually in a visually isolated compartment. Test compartments were approx. 12 m^2^ (ravens) and 25 m^2^ (keas). At the onset of a trial, the birds stayed in an observation room, watching the experimenter (E) in an adjacent presentation room through a closed wire mesh door. In tests involving ravens, the birds sat on the ground and E placed the tubes on the ground. The keas sat on a 1 m×1 m×1,2 m square table and the tubes were placed on an adjacent table of identical appearance in the presentation compartment. When entering the presentation compartment, the birds could step from the table in the observation compartment onto the table in the presentation compartment, ensuring that the setup was in sight of the birds throughout testing. The distance between the setup and the wire mesh door separating the two compartments was approx. 70 cm. One week prior to the start of each experiment, the birds were provided with the tubes to allow full habituation.

#### Tubes

**Figure 1:**
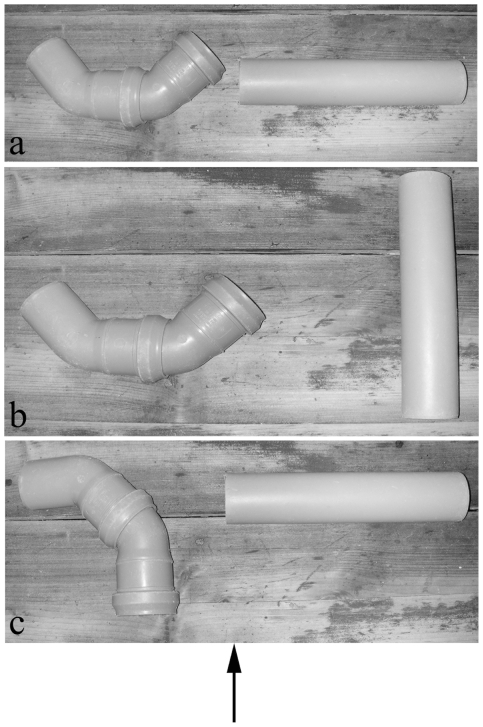
photos of the tubes used in experiment 1. Tubes are aligned as in a) “ST”-trials, b) “Straight+” and “Straight-“-trials and c) “Bent+” and “Bent-”trials. Black arrow indicates the birds' viewing angle. The distance between the tubes does not represent the original setting but has been reduced for demonstration purposes.

We used two grey, opaque PVC tubes of approx. 22 cm length and a diameter of 5 cm. One tube was straight, whereas the second tube had two 45° angles, preventing the birds from looking through the tube (Fig. 1).

#### Training

The birds received training sessions to familiarize them with having to choose one of the tubes. Therefore, E placed the two tubes simultaneously on the ground or on the table, respectively, positioning the openings of the tubes at a 90° angle away from the bird so that it could not see the content of either of the two tubes (Fig. 1a). The bent and straight tubes were positioned left or right randomly, with the exception that they were not placed on the same side consecutively more than twice. Then, E called the bird's name, showed the food reward to the bird and placed the reward (visible to the bird) in one of the two tubes. Next, E opened the door and the bird was allowed to approach both tubes and look into them. A choice was considered to have been made as soon as the bird touched one tube either with the beak or the foot, irrespective of whether it was the baited or the un-baited tube. A choice of the baited tube was considered as a success and the bird was allowed to retrieve the food; if it chose the un-baited tube, E stepped forward and removed both tubes to prevent the bird from obtaining the food. During the whole procedure, E stayed approx. 1.5 m behind the tubes looking straight ahead.

After the retrieval of the food, the bird returned to the observation room and the next trial started. The inter-trial interval was set to at least 20 sec, with the exact time dependant on the behaviour of the bird. Each training session consisted of 10 trials and a bird was advanced to testing if it chose the baited tube in 8 out of 10 trials in two consecutive sessions. One kea failed to reach the criterion and was omitted from further testing.

#### Testing

The birds were tested once per day. In contrast to the training trials, the food was hidden out of view of the birds in an adjacent room (ravens), or behind a barrier inside the presentation room (keas). After the baiting, E carried the tubes to the place of presentation and positioned the tubes simultaneously on the ground/table, with a distance of approx. 50 cm between the tubes. While carrying the tubes, E held the tubes horizontally, paying explicit attention that food did not move inside the tubes or could be seen by the bird.

After the positioning of the tubes, the bird could observe the setup for 3–5 seconds before E opened the door to allow the bird to choose one tube. If the bird chose the baited tube, it was allowed to retrieve the food and eat it. If it chose the un-baited tube, E stepped forward and removed both tubes, the subject returned to the observation room and E closed the wire mesh door. E then baited the tubes again and a new trial started. The birds received ten trials per session, with five different trial conditions being presented:

#### Standard trials (ST)

both tubes are positioned on the ground/table with the openings of the tubes turned 90° away from the bird; the content of the tubes is not visible (Fig. 1a).

#### Probe trials

In probe trials, one tube was turned by 90° compared to “ST”-trials, allowing the bird to see the content of the tube before approaching the setup (Fig. 1b, c). In total, four different probe trial conditions were applied:

#### Straight tube with food visible (Straight+)

Food was visible inside the straight tube

#### Bent tube with food visible (Bent+)

Food was visible inside the bent tube

#### Straight tube without food visible (Straight-)

The inside of the straight tube was visible, but the tube was empty. The food was positioned in the bent tube.

#### Bent tube without food visible (Bent-)

The inside of the bent tube was visible, but no food was visible. In 50% of the trials, the food was in the straight tube, in the other 50% of the trials the food was behind the bend inside the bent tube. We randomly chose whether food was inside the bent or the straight tube, with the exception that the food was not placed in the same tube more than twice in a row.

Per session, the birds received eight “ST”- trials and two randomly selected probe trials. We administered a total of 12 trials per probe trial condition and 192 “ST”- trials. However, some ravens refused to participate in some “ST”- trials and therefore received only 184 +/− 10.25 (×+/− SD) “ST”- trials. All keas participated on all trials.

We measured the following parameters:

We took success rate as an indicator of the overall performance of the birds in this task. Success rate was defined as the percentage of trials in which the birds chose the baited tube.

To assess in more detail how the birds solved the task, we analysed the strategies the birds used to find the food. Therefore, we measured

- rate of inspections, defined as looking into a tube before making a choice. We assessed an inspection if the birds approached a side of a tube and – before inserting the beak into the tube or grasping it – clearly paused in front of the tube and looked into it.

To assess if the birds would appreciate the impact of the shapes of the tubes on the visual access to the food we additionally measured:

- Timing of inspection: we distinguished between simultaneous inspections of both tubes and serial inspections. Simultaneous inspections were defined as approaching the tubes, slowing down in pace, standing in the middle, +/− equidistantly between both tubes and – by lowering the head – looking into both tubes +/− simultaneously (due to their laterally placed eyes, birds can easily look into both tubes at the same time). Note that simultaneous inspections were only possible in “ST” trials. Due to the position of the tubes (see Fig. 1), it was not possible to look into both tubes simultaneously when standing between the two tubes in probe trials. In serial inspections, the birds approached one tube at a time and looked into them consecutively. For serial inspections, the birds could also stand in the middle between the two tubes, but with their head clearly turned towards one tube at a time.

- Inspection pattern: for each tube, we distinguished whether the birds looked into it from one side or from both sides. Following our definition of looking (see above), a bird had to pause in front of the tube to look into it. Consequently, if, for example, a bird looked into one side of the tube, walked to the other side of the tube and instantaneously inserted the beak into the tube (or grasped it), we assessed this as looking into the tube from one side only.

Three instances were considered as indication that the birds relied on exclusion to choose a particular tube:

- in “Straight-” trials, if the birds chose the bent tube without prior inspection of any of the tubes (i.e., their choice behaviour would be similar to the chimpanzees' behaviour in [3], [20])
- in “Straight-” trials, if the birds chose the bent tube without looking into it, after having looked into the straight tube (i.e., their behaviour would be similar to the chimpanzees' behaviour in [14])
- in “ST”- trials, if the birds exhibited serial inspections, looked first into the empty tube and then chose the other tube without inspecting it (i.e., their behaviour would be similar to the chimpanzees' behaviour in [14]). Choices of the bent tube were not considered if the birds had looked into the straight tube from both sides previously, as these “redundant” looks appeared to be counter-intuitive to the concept of exclusion.

All sessions were videotaped and analysed from tape. Five sessions with ravens could not be recorded due to technical problems and had to be coded live. A second person not involved in this study but familiar with ravens and keas coded 50 trials per species to assess inter-observer reliability [48]. Inter -observer reliability was excellent (ravens: 94.57% concordance; Cohen's K = 0.94; keas: 98% concordance, Cohen's K = 0.98).

#### Predictions

We predicted that the birds would adjust their search behaviour according to their knowledge about the location of the food and that they would not inspect the tubes if they could see the food inside the tube before approaching it. If the birds appreciate the difference between the bent and the straight tube they should look into the bent tube more often than into the straight tube from both sides. Additionally, regarding EP, the birds should inspect the tubes in the “Bent-”-condition more frequently compared to the “Straight-”-condition and we would predict that in the “Straight-”condition, the birds would choose the bent tube without inspection. Additionally, in the “ST”-condition, the birds should choose a tube without inspecting it after having looked in the empty tube first.

More specifically, we made the following predictions for the different conditions:

#### Standard trials (ST)

The birds should inspect the tubes before making a choice (defined as the first touching of a tube), either by looking in both tubes simultaneously or in serial order.

#### Straight tube with food visible (Straight+)

The birds are expected to approach the straight tube directly and retrieve the food without prior inspection of any tube.

#### Bent tube with food visible (Bent+)

The birds are expected to approach the bent tube directly and retrieve the food without prior inspection of any tube.

#### Straight tube without food visible (Straight-)

The birds should avoid the straight tube and choose the bent tube without inspecting it first, i.e. their inspection behaviour should be similar to “Straight+” - and “Bent+” – trials.

#### Bent tube without food visible (Bent-)

The birds should inspect the tubes before choosing, i.e. their inspection behaviour should be similar to “ST” – trials.

### Experiment 2: Food hidden under bowls

#### Subjects

The same keas served as subjects. At the time of testing, not all of the ravens tested in experiment 1 were available. Therefore, we used a subset of five of the previously tested birds (two males, three females) and one additional nine year old male.

#### Testing procedure

The ravens were tested in Summer 2007 by AD, the tests with keas were conducted in Spring 2007 by CS. The general testing procedure was identical to experiment 1, with the exception that we used two equally looking plastic bowls (approx. 15 cm in diameter and 10 cm in height) instead of tubes.

#### Training

Prior to testing, the birds were familiarized with having to choose one of the bowls. E placed both bowls on a wooden board, with the board positioned on the ground (ravens) or on the table (keas) in front of the birds. The distance between the two bowls was approx. 40 cm, and the distance between the bowls and the bird was approx. 1 m, with the exact distance depending on the position of the bird (with a wire mesh door separating the bird and the bowls). Then, E called the bird's name and visible to the bird placed one piece of food under one of the two bowls. The food was positioned randomly on the left or on the right, with the exception that the food was not positioned on the same side consecutively more than twice in a row. Next, E opened the door and the bird was allowed to approach both bowls and lift one of them. If the bird chose the baited bowl, it was allowed to retrieve the food; if the bird attempted to approach the second bowl, E stepped forward and removed both bowls. During the whole procedure, E stayed approx. 1.5 m behind the bowls looking straight ahead. The next trial started as soon as the bird had returned to the observation room. The inter-trial interval was set to at least 20 sec, with the exact time dependant on the behaviour of the bird. Each training session consisted of 10 trials and the birds were advanced to testing if they chose the baited bowl in 8 out of 10 trials in two consecutive sessions. Two keas failed to reach the criterion and were omitted from further testing.

#### Testing

The birds received one session per day. For testing, the food was hidden out of view of the birds in an adjacent room (ravens), or behind a barrier inside the presentation room (keas). After the baiting, E carried the board with the bowls on top to the place of presentation. The distance to the wire mesh door separating the two compartments was approx. 70 cm.

After the positioning of the board, E called the bird's name and then provided one of four different cues:

#### Lifting both bowls

E touched both bowls with his arms extended and lifted the bowls to a height of approx. 40 cm above ground and then returned the bowls to the starting position.

#### Lifting the baited bowl

E touched both bowls, but lifted only the baited bowl, so that the food could be seen lying on the board. During presentation, E continued to touch the un-baited bowl.

#### Lifting the empty bowl

As before, with the exception that the empty bowl was lifted

#### Control

No bowl was lifted but both cups were touched by E.

Each cue lasted for 5 seconds and E looked straight ahead during the presentation of the cue. Then, E opened the wire mesh door and the bird was allowed to choose a bowl. If the bird chose the baited bowl, it was allowed to retrieve the food and eat it. If it chose the empty bowl, E stepped forward and removed both bowls. After the bird had returned to the observation room, E closed the wire mesh door and a new trial started.

The keas received six sessions, with twelve trials per session and three trials per condition. In an unrelated study conducted at the same time, the ravens apparently lost concentration if they received too many trials per session. Therefore, the ravens received eight sessions, with eight trials per session and two trials per condition. Since we do not have any indication that the keas lost their concentration during the course of a session, we consider both setups as equivalent.

Per trial, we measured whether the bird chose the baited or the un-baited bowl. All sessions with the keas and the sessions with three of the six ravens were videotaped and later analysed from tape. For technical reasons, video recording was not possible in case of the other three ravens and trials were coded live. As the choice of a bowl was unambiguous in any case, we did not calculate an inter-observer reliability.

### Analysis

We used the Kolmogorov-Smirnov procedure to test for deviation from normal distribution. We used Mann-Whitney-U-Tests to compare the ravens' and the keas' performance in the training conditions.

We tested for differences between conditions and species using two-way repeated measures ANOVAs as the most powerful procedure. We applied this procedure also in case of not normally distributed data, since ANOVA procedures are robust against violations of normal distribution [49], [50]. “Species” was used as between-subject factor and “condition” as within-subject factor. For post-hoc analysis, we preferred Holm-Sidak-tests over the more conservative Tukey-test to reduce the risk of committing a Type II – error based on our low sample size [51].

To assess if the birds' success rates differed from chance, we used Wilcoxon signed-rank tests or paired t-tests, as appropriate. To assess learning effects, we compared the performances in the first half and the second half of the experiments with Wilcoxon signed-rank tests or paired t-tests, as appropriate. In experiment 2, we additionally assessed whether performances increased over the course of the experiment, using Spearman signed-rank correlations.

All tests were conducted two-tailed and alpha was set to 0.05. Due to our relatively small sample size, we report exact P-values for all non-parametric tests [52]. Data analysis was conducted using SigmaStat 3.5 and SPSS for Windows 11.5.

## Results

### Experiment 1: Food Hidden in tubes

#### Training sessions

The ravens and the keas did not differ in the number of sessions they needed to reach the training criterion (ravens: 3 +/− 1.6 sessions (×+/−SD); keas: 3.3+/−0.87 sessions (×+/−SD); Mann-Whitney-U-Test: N = 17, U = 23.5, P = 0.236).

#### Success rate

**Figure 2:**
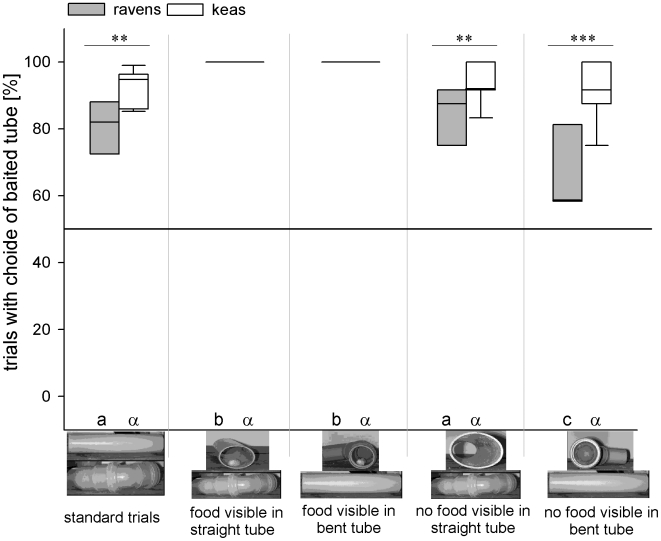
Percentage of trials in which the baited tube was chosen in the different conditions by ravens and keas. Box plots show median and upper and lower quartile. Whiskers indicate 10%- and 90% - confidence interval. Horizontal line indicates 50% chance level. Pictures below x-axis show the condition-specific tube arrangement. Between-species differences within conditions are indicated by asterisks (post-hoc Holm-Sidak analysis). Within-species differences between conditions are indicated by different letters below the bars. Roman letters (a, b, c) refer to comparisons within ravens, greek letters (α) refer to comparisons within keas. Bars marked with different letters differ significantly from each other (post-hoc Holm-Sidak analysis).

The ravens and the keas chose the baited tube above chance level in all five conditions (Wilcoxon or paired t-test, as appropriate: in all cases P<0.001). A two-way repeated measure ANOVA revealed significant differences between the two species (F_1,15_ = 18.386, P<0.001), conditions (F_4,60_ = 23.914, P<0.001), and a significant species×condition - interaction (F_4_,_60_ = 7.756, P<0.001). Within-species post-hoc comparisons (Holm-Sidak tests; for exact significance levels see Appendix S1) revealed that the ravens were significantly more successful when they saw the food before approaching the tubes (conditions “Bent+” and “Straight+”), compared to all other conditions. The significantly lowest success rate was found if the birds saw the bent tube without food before approaching (condition “Bent-”; Fig. 2). In contrast, the keas' success did not differ between conditions (see Appendix S1).

Within-condition post-hoc comparisons (Holm-Sidak tests) revealed that in “ST”-trials, the keas were more successful than the ravens (t = 2.82, P = 0.006). If food was visible before approaching (“Bent+” and “Straight+”), no significant differences between the ravens and the keas could be detected (both conditions: t<0.001, P>0.999). In contrast, in the “Straight-” -condition (t = 3.077, P = 0.003) and in the “Bent-” -condition (t = 6.183, P<0.001), the keas chose the baited tube significantly more often than the ravens. Consequently, overall, the keas chose the baited tube more often than the ravens (paired t-test: N = 17, t = −3.041, P = 0.008).

The performance of the ravens did not change over the course of the experiment (all conditions: paired t-test or Wilcoxon-test, as appropriate: N  = 8, P≥0.126). In contrast, the keas chose the baited tube more often in the second half than in the first half of the experiment in “ST”-trials (paired t-test: N = 9, t = −4.252, P = 0.003), but no difference was detected in any of the probe-trial conditions (all conditions: paired t-test or Wilcoxon-test, as appropriate: N  = 9, P≥0.063).

#### Inspections

**Figure 3:**
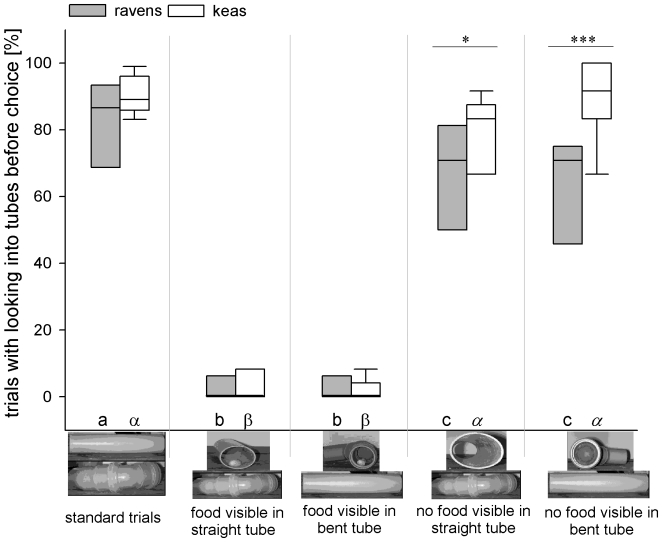
Percentage of trials in which birds inspected at least one tube before making a choice. Box plots show median and upper and lower quartile. Whiskers indicate 10%- and 90% - confidence interval. Pictures below x-axis show the condition-specific tube arrangement. Between-species differences within conditions are indicated by asterisks (post-hoc Holm-Sidak analysis). Within-species differences between conditions are indicated by different letters below the bars. Roman letters (a,b,c) refer to comparisons within ravens and greek letters (α, β, γ) refer to comparisons within keas. Bars marked with different letters differ significantly from another (post-hoc Holm-Sidak analysis), letters printed in italics indicate a trend to differ from bars marked with normally printed letters.

These high success rates (above), even in conditions in which the birds could not see the location of the food at the start of a trial, were due to the high inspection rates (Fig. 3). This is further supported by our finding that in both species, individuals were more successful when they inspected tubes than when they did not (both Wilcoxon sign-rank test: ravens: N = 8; Z = −2.366; P = 0.008; keas: N = 9, Z = −2.666; P = 0.004).

A two-way repeated measure ANOVA with inspection rate as dependent factor revealed significant differences between the two species (F_1,15_ = 7.289, P = 0.016), conditions (F_4,60_ = 332.066, P<0.001), as well as a significant species×condition - interaction (F_4_,_60_ = 6.719, P<0.001). Within-species post-hoc comparison (Holm-Sidak test, for exact statistical analysis see Appendix S2) revealed that the ravens hardly ever inspected tubes if they saw the food before they approached the tubes (conditions “Straight+” and “Bent+”), but they inspected both tubes significantly more often if they saw the inside of a tube without food (conditions “Straight-” and “Bent-”). However, they showed the significantly highest inspection rates if they did not see the content of any of the tubes (“ST”; see also Fig. 3).

Similarly, the keas hardly ever inspected the tubes if they had already seen the food before approaching. In contrast to the ravens, the keas were equally likely to inspect the tubes in the “ST” and “Bent-” - conditions. In the “Straight-” - condition, the keas inspected the tubes less frequently than in “ST” -and “Bent-” - trials, even though these comparisons marginally failed to reach significance (Appendix S2; Fig. 3).

Comparisons between the two species revealed that the inspection rates did not differ between ravens and keas in “ST”- (Holm-Sidak: t = 1.473, P = 0.147), “Straight+”- (Holm-Sidak: t = 0.441, P = 0.661) and “Bent+”-trials (Holm-Sidak: t = 0.04, P = 0.968), but the ravens inspected the tubes less frequently than the keas in “Straight-”- (Holm-Sidak: t = 2.604, P = 0.012) and “Bent-”-trials (Holm-Sidak: t = 5.087, P<0.001).

When comparing the first and the second half of the experiment, we found no change in the ravens' performance in any condition (Wilcoxon-test or paired t-test, as appropriate: all N = 8; all P>0.165). The keas increased their inspection rates over the course of the experiment in “ST” – trials (paired t-test: N = 9, t = −2.337, P = 0.048), but did not change inspection rates in any of the other conditions (Wilcoxon-test or paired t-test, as appropriate: all N = 9; all P>0.437).

#### Timing of inspections

**Figure 4:**
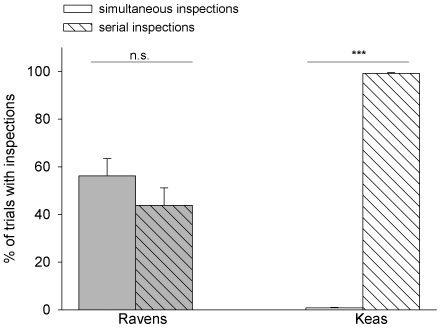
Frequency of simultaneous and serial inspections in “ST-trials”, given as percentage of all trials in which birds inspected at least one tube before making a choice. Bars show the mean, whiskers indicate SE. Statistical information refers to paired t-tests.

If the ravens inspected the tubes in “ST”- trials, they were equally likely to inspect both tubes simultaneously as to inspect one tube at a time (paired t-test: N = 8, t = 0.849, df = 7, P = 0.424), whereas the keas nearly exclusively showed serial inspections (paired t-test: N = 9, t = −174.531, df = 8, P<0.001; see also Fig. 4). Over the course of the experiment, the ravens increased the rate of simultaneous inspections (paired t-test: N = 8, t = −5.487, df = 7, P<0.001), whereas the keas did not (Wilcoxon-test: N = 9, T^+^ = 8, P = 0.688).

#### Inspection patterns

**Figure 5:**
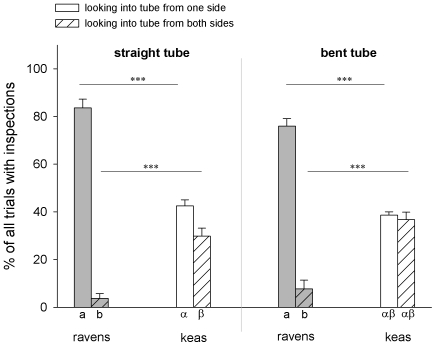
Frequency of looking into a tube from one side or both sides. Bars show mean and SE. Within-species differences between conditions are indicated by different letters below bars. Roman letters (a,b) refer to comparisons within ravens and greek letters (α,β) refer to comparisons within keas. Bars marked with different letters differ significantly (post-hoc Holm-Sidak analysis).

A two-way repeated measures ANOVA revealed significant effects of species (F_1,15_ = 11.236, P = 0.004), inspection type (F_3,45_ = 118.092, P<0.001) and species×inspection type – interaction (F_3,45_ = 78.273, P<0.001). Post-hoc Holm-Sidak analyses revealed that the ravens were more likely to look into a tube from one side than from both sides (straight tube, looking once vs. twice: t = 17.794, P<0.001; bent tube, looking once vs. twice: t = 15.200, P<0.001). Similarly, the keas were more likely to look into the straight tube from one side only (t = 2.988, P = 0.005), but were equally likely to look into the bent tube from one side as from both sides (Holm-Sidak: t = 0.458, P = 0.649). Between species comparisons reveal that the ravens looked into both tubes from one side more frequently than the keas (straight tube: t = 9.894, P<0.001; bent tube: t = 8.962, P<0.001). Logically, the keas looked into both tubes from both sides more often than ravens (straight tube: t = 6.273, P<0.001; bent tube: t = 6.979, P<0.001; Fig. 5).

Comparisons between the first and the second half of the experiment revealed that the ravens did not change their inspecting behaviour (paired t-tests, all P>0.11). In contrast, the keas decreased the frequency of looking into the straight tube from both sides over the course of the experiment (paired t-test: N = 9, t = 2.472, df = 8, P = 0.039) and increased the frequency of looking into the bent tube from both sides (paired t-test: N = 9, t = −3.675, df = 8, P = 0.006).

#### Exclusion

**Figure 6:**
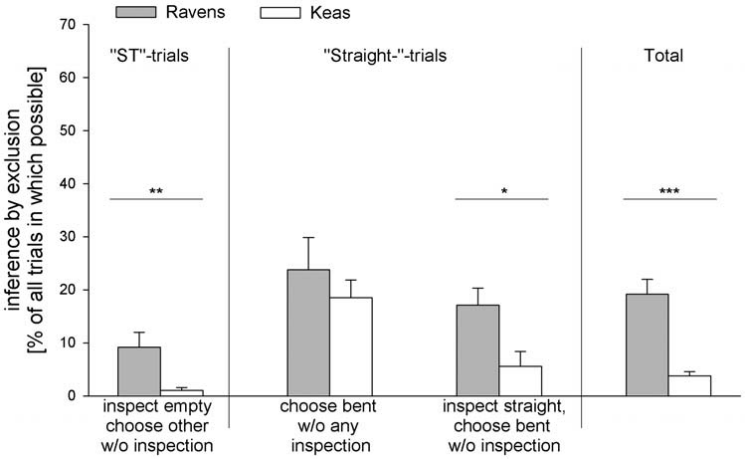
Occurrence of tube-choices which may be based on “inference by exclusion”. Bars show mean, whiskers indicate SE. Statistical information refers to Mann-Whitney-U-tests and t-tests, respectively.

Throughout the experiment, we found instances of exclusion in both species. In “ST”- trials, the ravens and the keas infrequently chose a tube without prior inspection after inspecting the empty tube first, with the ravens showing this choice pattern more frequently than keas (t-test: N = 17, t = 2.977, df = 15, P = 0.009; Fig. 6). In “Straight-” - trials, the ravens and the keas were equally likely to choose the baited tube without prior inspection of any of the two tubes (t-test: N = 17, t = 0.776, df = 15, P = 0.45); however, if looking first into the empty tube in “Straight-”-trials, the ravens were more likely than the keas to choose the baited tube without inspecting it first (Mann-Whitney-U-test: N_1_ = 8, N_2_ = 9, U = 11, P = 0.015). Taking all these instances together, the ravens chose by exclusion in 19.17% of all trials in which this was possible (i.e. in “Straight-”- trials and “ST ”- trials with serial inspections, in which the empty tube was inspected first), whereas the keas showed such choices in only 3.78% of trials (t-test: N = 17, t = 5.535, df = 15, P<0.001; Fig. 6). No change in performance was detectable over the course of the experiment (ravens: “ST”- trials: paired t-test: N = 8, t = 1.624, df = 7, P = 0.148; “Straight-”- trials, direct choice of bent tube: paired t-test: N = 8, t = −0.704, df = 7, P = 0.504; “Straight-”- trials, choice of bent tube after inspecting straight tube: paired t-test: N = 8, t = 0.683, df = 7, P = 0.516; keas: “ST”- trials: Wilcoxon-test: N = 9, T^+^ = 5, P>0.999; “Straight-”- trials, direct choice of bent tube: paired t-test: N = 9, t = −0.883, df = 8, P = 0.403; “Straight-”- trials, choice of bent tube after inspecting straight tube: Wilcoxon-test: N = 9, T^+^ = 7, P = 0.625).

### Experiment 2: Food hidden under bowls

#### Training sessions

The ravens and the keas did not differ significantly in the number of sessions needed to reach the training criterion (ravens: 3.33+/−1.21 sessions (x+/−SD); keas: 2.57+/−1.13 sessions (×+/−SD); Mann-Whitney-U-Test: N = 13; U = 13; P = 0.295).

#### Test sessions

**Figure 7:**
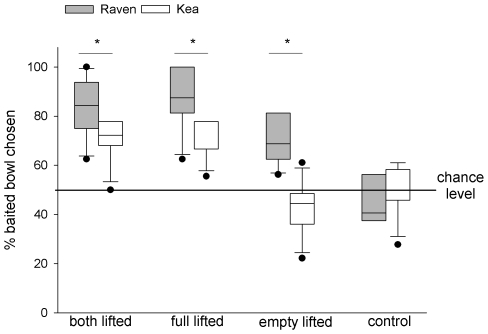
Percentage of trials in which the baited bowl was chosen in Experiment 2. Box plots show median and upper and lower quartile. Whiskers indicate 10%- and 90% - confidence interval. Between-species differences are indicated by asterisks (post-hoc Holm-Sidak test).

A two-way repeated measures ANOVA revealed a significant effect of species (F_1,11_ = 14.396, P = 0.003), condition (F_3,33_ = 26.436, P<0.001) and species×condition−interaction (F_3,33_ = 4.718, P = 0.008). Post-hoc analysis revealed that the ravens chose the baited bowl significantly more often in all test-conditions when compared to the control condition (Holm-Sidak tests with N = 6; both bowls lifted: t = 6.184, P<0.001; baited bowl lifted: t = 6.685, P<0.001, empty bowl lifted: t = 4.011, P<0.001). In contrast, compared to the control trials, the keas chose the baited bowl significantly more often only when the food could be seen during cueing (Holm-Sidak tests with N = 7; both bowls lifted: t = 3.713, P = 0.001; baited bowl lifted: t = 3.576, P = 0.001), but not if the empty bowl was lifted (Holm-Sidak test: N = 7, t = 1.1, P = 0.279). Additional comparisons revealed that the ravens chose the baited bowl significantly more often than the keas in all three test conditions (Holm-Sidak - tests with N = 13; both bowls lifted: t = 2.041, P = 0.047; baited bowl lifted: t = 2.67, P = 0.011, empty bowl lifted: t = 4.328, P<0.001) but not in the control condition (Holm-Sidak - test: N = 13, t = 0.709, P = 0.482; see Fig. 7).

As the ravens performed above chance in the condition with the empty bowl lifted, we further investigated the possibility that the birds may have learned where to find the food in this particular condition: the ravens' performance did not differ between the first and the last session (paired T-test: N = 6, t = 0, df = 5, P>0.999) of the experiment. When comparing the performance in the first four sessions of the experiment with the last four sessions of the experiment, the ravens' performance increased from a mean of 62.5% of the trials correct in the first half to a mean of 75% in the second half of the experiment (paired t-test: N = 6; t = −3.873; df = 5, P = 0.012). However, there was no continuous increase in performance (Spearman rank correlation: r_s_ = 0.331, P = 0.423).

On an individual level, only one (out of six birds) performed below 50% in the first half of the experiment: after being correct on 37.5% of the trials in the first half, it was correct on 62.5% of the trials in the second half of the experiment. When excluding this bird from the analysis, the other five birds were correct on a mean of 67.5% of the trials in the first four sessions, which is significantly better than chance (Wilcoxon signed-rank-test: N = 5, Z—2.070, P = 0.038). During the last four sessions, these five birds were correct on a mean of 77.5% of the trials (Wilcoxon signed-rank test: N = 5, Z = −2.041, P = 0.041).

## Discussion

We tested two distantly related, but similarly large-brained bird species [29] in two choice tasks, in which food had to be found in one of two possible locations. Although both species have demonstrated advanced cognitive abilities in a variety of tasks (e.g. [57]–[60]), they performed differently in our study, as we found solid evidence for exclusion [3] in ravens only.

Experiment 1 was solved with ease by both species, as they chose the baited tube at high levels. Additionally, they based their search behaviour on their previously acquired knowledge, i.e. if they had seen the food, both species directly approached the tube to retrieve the food, but they inspected the tubes if they did not know where food was to be found. Furthermore, when they saw an empty tube, both species reduced the number of inspections. The search pattern of the ravens in these conditions (“Straight-” and “Bent-”) differed from the “ST”- condition, in which they neither saw nor could know where the food was hidden.

Similar findings in chimpanzees and rhesus monkeys *Macaca mulatta* have been interpreted as an indication of meta-cognition, i.e. “knowing what is known” [14], [16]. This interpretation has been criticized, as the increased searching in case of ignorance about the hiding place could be explained alternatively by an internal state of uncertainty, without any need for meta-knowledge [18]. Therefore, it remains unclear if the ravens and the keas were aware of their current state of knowledge. Noteworthy, capuchin monkeys tested in a similar experiment did not reduce their search effort [17], suggesting possible species differences either in information processing or in the tendency to inhibit search behaviour.

Most importantly, the ravens and the keas differed strikingly in the way they searched: the ravens frequently approached the setup and inspected both tubes at the same time. Usually, this first look was sufficient for them to make their choice, as the ravens looked into a tube from both sides only infrequently. On the contrary, the keas appeared to be the more thorough explorers, as they often looked into both tubes from both sides. Such double inspections may be useful when looking into the bent tube, but may be regarded as redundant [17] when inspecting the straight tube. Over the course of the experiment, the keas adjusted their search behaviour and reduced the number of double-inspections of the straight tube while they increased the rate of double inspections of the bent tube. Still, the number of double inspections of the straight tube remained high until the end of the experiment (approx. 27% of all trials). In sum, this suggests that the keas may not have appreciated the impact of the shape of the tubes on the visual access to the food. Alternatively, efficiency in foraging may be of less importance for keas than for ravens. Although the latter did not show such redundant searches, our results still do not suggest that the ravens understood the difference between the tubes. In the “Straight-” and “Bent-” – conditions, the ravens treated both tubes equally and reduced their search effort in both conditions, even though searching would have been required in the “Bent-” - condition. This may appear puzzling, as ravens follow gaze geometrically and may understand how a barrier may block their own line of sight [43], [53]. However, children can use barriers as a screen for getting out of sight when three years old [54], but do not understand the linearity of a line of sight until they are five years old [19].

The reduced search effort in the “Straight-”-condition particularly in ravens suggests that they may have been aware of the location of the food without having seen it before. This is further supported by choices indicating EP, again particularly in the ravens but to a lesser extend also in the keas. However, how they came to choose by exclusion is unclear. On the one hand no change in performance was detectable during the course of the experiment, arguing against a learned response. On the other hand, this experiment required extensive training procedures and none of the birds was naïve to experimental testing, so that learning may have occurred before the start of testing. Still, as the birds did not apply any of the potential search strategies consistently in the “Straight-”-condition (see also Fig. 6), a conditioned response seems to be unlikely and both avoidance and inference by exclusion remain possible mechanisms underlying the birds' choices.

We are confident that the birds' EP was not influenced by any food-related cues, i.e. odour. First, if smell guided the birds' behaviour, it is not conceivable why the inspection patterns differed between the conditions and why the keas, in particular, showed these high inspection rates. Second, birds are known to be insensitive to and usually unable to detect hidden food by smell [55]. Finally, several experiments convincingly demonstrated that ravens do not detect hidden food by smell [44], [45], [56], [57].

While the ravens demonstrated EP more frequently than the keas, the rates of exclusion obtained in this first experiment are still low. Critics may argue that this may be seen as a rather low evidence for EP. However, these rates resemble those found in other studies: Call and Carpenter confronted chimpanzees and human children with food hidden in two straight tubes. If they looked in the empty tube first, the chimpanzees chose the other tube in about 24% of the cases without prior inspection, compared to approx. 19% in ravens; the lower numbers for the keas (approx. 4% of all possible trials) resemble those of 2 ½ year old human children [14]; while these low EP rates in children may indicate an inability to choose by exclusion at that age [14], chimpanzees have been shown repeatedly to be capable of EP [2], [3], [8], [20], [25]. This suggests that the low rates of EP in these tube-experiments are not due to a lack of exclusion abilities. Rather, the setup used by Call & Carpenter [14] and in the current study may have contributed to the low EP-rates, as the subjects were not required to exclude one location in order to solve the task. Additionally, the keas were more successful than the ravens, suggesting that relying on exclusion is costly as it may increase the risk of errors. Experiment 2 was designed to investigate further whether ravens and keas do indeed differ in their reliance on exclusion, as in this experiment the birds were forced to choose by exclusion.

Interestingly, here the ravens clearly outperformed the keas and were more successful in all three test conditions. More importantly, if only the empty bowl was lifted during cue-presentation, only the ravens chose the baited bowl significantly above chance level. Again, the mechanism the ravens relied on to solve this task remains unclear. We found some indication of an improvement over the course of the experiment, but the ravens' performance did not increase continuously and five of the six birds chose the correct bowl from the beginning of the experiment. Hence, the ravens may have learned to solve the task very fast, but both an avoidance strategy [4] and inference by exclusion [3] are possible alternative explanations.

Importantly from a comparative point of view, the keas and the ravens differed in their performance, as the keas did not choose by exclusion. Hence, irrespective of the cognitive mechanism applied by the ravens, the keas apparently did not rely on this mechanism in the same way as the ravens did.

From an evolutionary point of view, the differences between the two species suggest an adaptive specialisation according to the specific problems of the species' socio-ecological environment [12], [32], [61]. However, it is premature to draw final conclusions based on the performances in two closely related experiments only. For comparative research, it is crucial to choose test paradigms that are equally salient for all species to be compared [62]. We believe our setup was sufficiently straight-forward and general enough to be equally fair for both species, but if this is the case, why do ravens use exclusion more readily than keas?

One of the most salient differences between the two species is that ravens regularly cache food and pilfer others' food caches [56], [63], while keas (like all parrots) do not cache at all. Therefore, ravens, much more than keas, are characterized by competition over cached items. Ape-like deceptive behaviours such as fake-caching and misleading are well documented in ravens [57], [64], while keas are known primarily for their extreme object manipulation and play behaviour [47], [59], [65]–[69].

Food caching has been shown to have a major impact on animals' cognitive abilities [61], [62], [70]–[76]. For example, differences in spatial memory seem to exist between food-storing and non-storing species [71], [77]; food storing tits and corvids selectively return to places where food had been found before, whereas non-storing tits and corvids selectively return to places where they have been before, irrespective of whether they had found food there [77], [78]. This suggests differences in the memory system of storers and non-storers but also raises the question of whether storers are more effective than non-storers in avoiding empty food locations.

In the presence of dominant competitors, ravens avoid approaching the caches made by these individuals [56] and when competing with other pilferers over caches, they selectively and rapidly pilfer caches that the other is aware of, but avoid caches the other is ignorant about [58], [79]. These results highlight two important characteristics of raven behaviour: first, depending on the social context, they inhibit their tendency to pilfer food caches; secondly, speed matters when competing with others over caches. For keas, neither inhibition nor speed may be of major importance; rather, the extreme neophilia and the intense play behaviour of keas may lead to exactly the opposite pattern and prompt them to devote more time to object manipulation and exploration.

In our experiments, this may explain why the keas searched more thoroughly, inspected both tubes from both sides more often and demonstrated less exclusion than the ravens. In contrast, the ravens may have been primed to increase speed by avoiding redundant inspections and using exclusion.

However, the question remains why the keas did not demonstrate EP in experiment 2, when they were forced to do so. Keas may lack the cognitive prerequisites to show exclusion instantaneously and the number of trials may have been too low for them to learn to solve the task. An alternative interpretation could be that the keas did not choose by exclusion because their choice behaviour is guided by different cues compared to ravens. Dogs tested in a task similar to our second experiment did not choose the baited bowl when they saw the empty bowl being lifted. In a second step, the food was hidden under one bowl which was then covered by a larger, outer bowl. This modification allowed the lifting of both outer bowls while the reward was still covered underneath the inner bowl. In this condition, dogs were able to solve the task, suggesting that dogs' “reasoning abilities are masked by the bias towards following social cues” ([5] p.735); in other words, the lifting of the bowls was more salient than the information about the food location. A similar result was obtained for dogs and chimpanzees with an array of different tests [25], in which the dogs responded more strongly than the chimpanzees to socially facilitated cues, whereas the chimpanzees were more successful than the dogs in tasks in which causal information was provided (i.e. inference tasks). Taken together, this led to the suggestion that the behaviour of dogs “may be described by a set of hierarchically ordered choice-rules and different combinations of these rules which can be used by the dog to make a decision in the problem situation. Using reasoning by exclusion is one of the competing possibilities, though not the dominant one, in the case of the dog” ([5], p.736). If this concept is applied to the data so far available, exclusion may be a higher-ranking problem-solving strategy in chimpanzees and ravens but may be lower-ranking in dogs and keas. Indeed, similar to dogs, keas are highly responsive to social cues and this may even overshadow the use of causal cues (Lichtenegger & Gajdon, unpubl. data) and the stronger cueing of the empty bowl in experiment 2 may have prevented them from inferring the correct food location.

In conclusion, our results clearly demonstrate different performances between ravens and keas in two exclusion experiments, suggesting that the cognitive evolution of corvids and parrots led at least to a different use of cognitive abilities in ravens and keas. We believe that food-storing may provide a useful framework to explain these results, but more species need to be incorporated to specify whether the current findings are due to differences between food-storing and non-storing species or differences between corvids and parrots.

## Supporting Information

Appendix S1 Success rate: test statistics for comparison of success rates between conditions and animal groups (Holm-Sidak procedure; overall significance level = 0.05). Note that for reasons of comparison, all tables list pair-wise comparisons in the same order
(0.06 MB DOC)

Appendix S2 Inspection rate: test statistics for comparison of inspection rates between conditions and animal groups (Holm-Sidak procedure; overall significance level = 0.05). Note that for reasons of comparison, all tables list pair-wise comparisons in the same order
(0.05 MB DOC)
