# Supplementary material for: What You See Is What You Get? Exclusion Performances in Ravens and Keas
Source: PLoS One. 2009 Aug 5;4(8):e6368. doi: 10.1371/journal.pone.0006368 (PMC2715862; doi:10.1371/journal.pone.0006368)
Supplement: Appendix S1 — Success rate: test statistics for comparison of success rates between conditions and animal groups (Holm-Sidak procedure; overall significance level = 0.05). Note that for reasons of comparison, all tables list pair-wise comparisons in the same order (0.06 MB DOC) [file pone.0006368.s001.doc]

Appendix 1: Success rate: test statistics for comparison of success rates between conditions and animal groups (Holm-Sidak procedure; overall significance level = 0.05). Note that for reasons of comparison, all tables list pair-wise comparisons in the same order

a) Comparison between conditions for ravens

| Condition comparison | T | Unadjusted P | Critical Level | Significance |
| --- | --- | --- | --- | --- |
| „ST“ vs. „Straight+“ | 5.035 | <0.001 | 0.006 | Yes |
| „ST “ vs. „Bent+“ | 5.035 | <0.001 | 0.007 | Yes |
| „ST“ vs. „Straight-“ | 0.791 | 0.432 | 0.025 | No |
| „ ST“ vs. „Bent-“ | 3.734 | < 0.001 | 0.017 | Yes |
| „Straight+“ vs. „Bent+“ | 0 | >0.999 | 0.050 | No |
| „Straight+“ vs. „Straight-“ | 4.243 | < 0.001 | 0.010 | Yes |
| „Straight+“ vs. „Bent-“ | 8.769 | < 0.001 | 0.005 | Yes |
| “Bent+” vs. “Straight-“ | 4.243 | <0.001 | 0.013 | Yes |
| „Bent+“ vs. „Bent-“ | 8.769 | < 0.001 | 0.006 | Yes |
| „Straight-“ vs. „Bent-“ | 4.526 | < 0.001 | 0.009 | Yes |

b) Comparison between conditions for keas

| Comparison | t | Unadjusted P | Critical Level | Significance |
| --- | --- | --- | --- | --- |
| „ST“ vs. „Straight+“ | 2.193 | 0.032 | 0.006 | No |
| „ST “ vs. „Bent+“ | 2.193 | 0.032 | 0.007 | No |
| „ST“ vs. „Straight-“ | 1.126 | 0.265 | 0.010 | No |
| „ ST“ vs. „Bent-“ | 0.207 | 0.836 | 0.025 | No |
| „Straight+“ vs. „Bent+“ | <0.001 | >0.999 | 0.050 | No |
| „Straight+“ vs. „Straight-“ | 1.067 | 0.290 | 0.013 | No |
| „Straight+“ vs. „Bent-“ | 2.4 | 0.020 | 0.005 | No |
| “Bent+” vs. “Straight-“ | 1.067 | 0.290 | 0.017 | No |
| „Bent+“ vs. „Bent-“ | 2.4 | 0.020 | 0.006 | No |
| „Straight-“ vs. „Bent-“ | 1.33 | 0.187 | 0.009 | No |
