# Supplementary material for: What You See Is What You Get? Exclusion Performances in Ravens and Keas
Source: PLoS One. 2009 Aug 5;4(8):e6368. doi: 10.1371/journal.pone.0006368 (PMC2715862; doi:10.1371/journal.pone.0006368)
Supplement: Appendix S2 — Inspection rate: test statistics for comparison of inspection rates between conditions and animal groups (Holm-Sidak procedure; overall significance level = 0.05). Note that for reasons of comparison, all tables list pair-wise comparisons in the same order (0.05 MB DOC) [file pone.0006368.s002.doc]

Appendix 2: Inspection rate: test statistics for comparison of inspection rates between conditions and animal groups (Holm-Sidak procedure; overall significance level = 0.05). Note that for reasons of comparison, all tables list pair-wise comparisons in the same order

a) Comparison between conditions for ravens

| Condition comparison | t | Unadjusted P | Critical Level | Significance |
| --- | --- | --- | --- | --- |
| „ST“ vs. „Straight+“ | 17.064 | < 0.001 | 0.005 | Yes |
| „ST “ vs. „Bent+“ | 17.064 | < 0.001 | 0.006 | Yes |
| „ST“ vs. „Straight-“ | 3.742 | < 0.001 | 0.017 | Yes |
| „ ST“ vs. „Bent-“ | 4.630 | < 0.001 | 0.013 | Yes |
| „Straight+“ vs. „Bent+“ | < 0.001 | > 0.999 | 0.050 | No |
| „Straight+“ vs. „Straight-“ | 13.323 | < 0.001 | 0.006 | Yes |
| „Straight+“ vs. „Bent-“ | 12.434 | < 0.001 | 0.009 | Yes |
| “Bent+” vs. “Straight-“ | 13.323 | < 0.001 | 0.007 | Yes |
| „Bent+“ vs. „Bent-“ | 12.434 | < 0.001 | 0.010 | No |
| „Straight-“ vs. „Bent-“ | 0.888 | 0.378 | 0.025 | No |

c) Comparison between conditions for keas

| Condition comparison | t | Unadjusted P | Critical Level | Significance |
| --- | --- | --- | --- | --- |
| „ST“ vs. „Straight+“ | 19.448 | < 0.001 | 0.006 | Yes |
| „ST “ vs. „Bent+“ | 20.076 | < 0.001 | 0.005 | Yes |
| „ST“ vs. „Straight-“ | 11.016 | 0.016 | 0.013 | No |
| „ ST“ vs. „Bent-“ | 0.188 | 0.852 | 0.050 | No |
| „Straight+“ vs. „Bent+“ | 0.628 | 0.532 | 0.025 | No |
| „Straight+“ vs. „Straight-“ | 16.957 | < 0.001 | 0.010 | Yes |
| „Straight+“ vs. „Bent-“ | 19.260 | < 0.001 | 0.007 | Yes |
| “Bent+” vs. “Straight-“ | 17.585 | <0.001 | 0.009 | Yes |
| „Bent+“ vs. „Bent-“ | 19.888 | <0.001 | 0.006 | Yes |
| „Straight-“ vs. „Bent-“ | 2.303 | 0.025 | 0.017 | No |
